# *Apc*^*Min/+*^ tumours and normal mouse small intestines show linear metabolite concentration and DNA cytosine hydroxymethylation gradients from pylorus to colon

**DOI:** 10.1038/s41598-020-70579-w

**Published:** 2020-08-12

**Authors:** Basetti Madhu, Santiago Uribe-Lewis, Martin Bachman, Adele Murrell, John R. Griffiths

**Affiliations:** 1 Cancer Research UK Cambridge Institute, University of Cambridge, Robinson Way, Cambridge, CB2 0RE UK; 2 Discovery Science and Technology, Medicines Discovery Catapult, Alderley Park, Macclesfield, SK10 4TG UK; 3 Centre for Regenerative Medicine, Department of Biology and Biochemistry, University of Bath, Bath, BA2 7AY UK

**Keywords:** Molecular biology, Oncology, Biochemistry, Metabolomics

## Abstract

Topographical variations of metabolite concentrations have been reported in the duodenum, jejunum and ileum of the small intestine, and in human intestinal tumours from those regions, but there are no published metabolite concentrations measurements correlated with linear position in the mouse small intestine or intestinal tumours. Since DNA methylation dynamics are influenced by metabolite concentrations, they too could show linear anatomical variation. We measured metabolites by HR-MAS ^1^H NMR spectroscopy and DNA cytosine modifications by LC/MS, in normal small intestines of C57BL/6J wild-type mice, and in normal and tumour samples from *Apc*^*Min/*+^ mice. Wild-type mouse intestines showed approximately linear, negative concentration gradations from the pylorus (i.e. the junction with the stomach) of alanine, choline compounds, creatine, leucine and valine. *Apc*^*Min/*+^ mouse tumours showed negative choline and valine gradients, but a positive glycine gradient. 5-Hydroxymethylcytosine showed a positive gradient in the tumours. The linear gradients we found along the length of the mouse small intestine and in tumours contrast with previous reports of discrete concentration changes in the duodenum, jejunum and ileum. To our knowledge, this is also the first report of a systematic measurement of global levels of DNA cytosine modification in wild-type and *Apc*^*Min/+*^ mouse small intestine.

## Introduction

Several recent studies have shown topographical variations of metabolite concentrations in the intestines of normal humans^1^, rats^2,3^ and mice^4^, and also in human tumours arising in different intestinal regions^5^. These reports led us to investigate whether mouse intestinal cancer metabolism may also depend on the anatomical location of the tumour in the small intestine of the *Apc*^*Min/*+^ mouse, a widely-used model of gut tumorigenesis that spontaneously develops adenomas in the small and large intestines^6^ which can be sampled from precise anatomical locations. This model was used to assess metabolic variations in both tumour and non-tumour tissues by HR-MAS ^1^H NMR^7^. The effect of anatomical location on metabolism of Apc^*Min/*+^ adenomas has not been investigated.

We therefore performed HR-MAS ^1^H NMR on biopsies taken along the length of the small intestines from wild-type C57BL/6J mice and *Apc*^*Min/*+^ littermates. HR-MAS ^1^H NMR is less sensitive than mass spectrometry (hence the limited number of metabolites analysed herein) but it has the advantage that metabolites are assayed in the original sample, with no errors due to extraction, derivatisation or ionisation; also, the sample is unaffected by the spectroscopic procedure, so the metabolite data can be paired to data from additional analyses. Because epigenetic changes associate with gene activity^8^, cellular proliferation^9,10^ and cancer cell metabolism^11^ we measured cytosine methylation and hydroxymethylation in DNA extracted from the tissue biopsies that had been used for the metabolite analysis.

## Results

**Figure 1:**
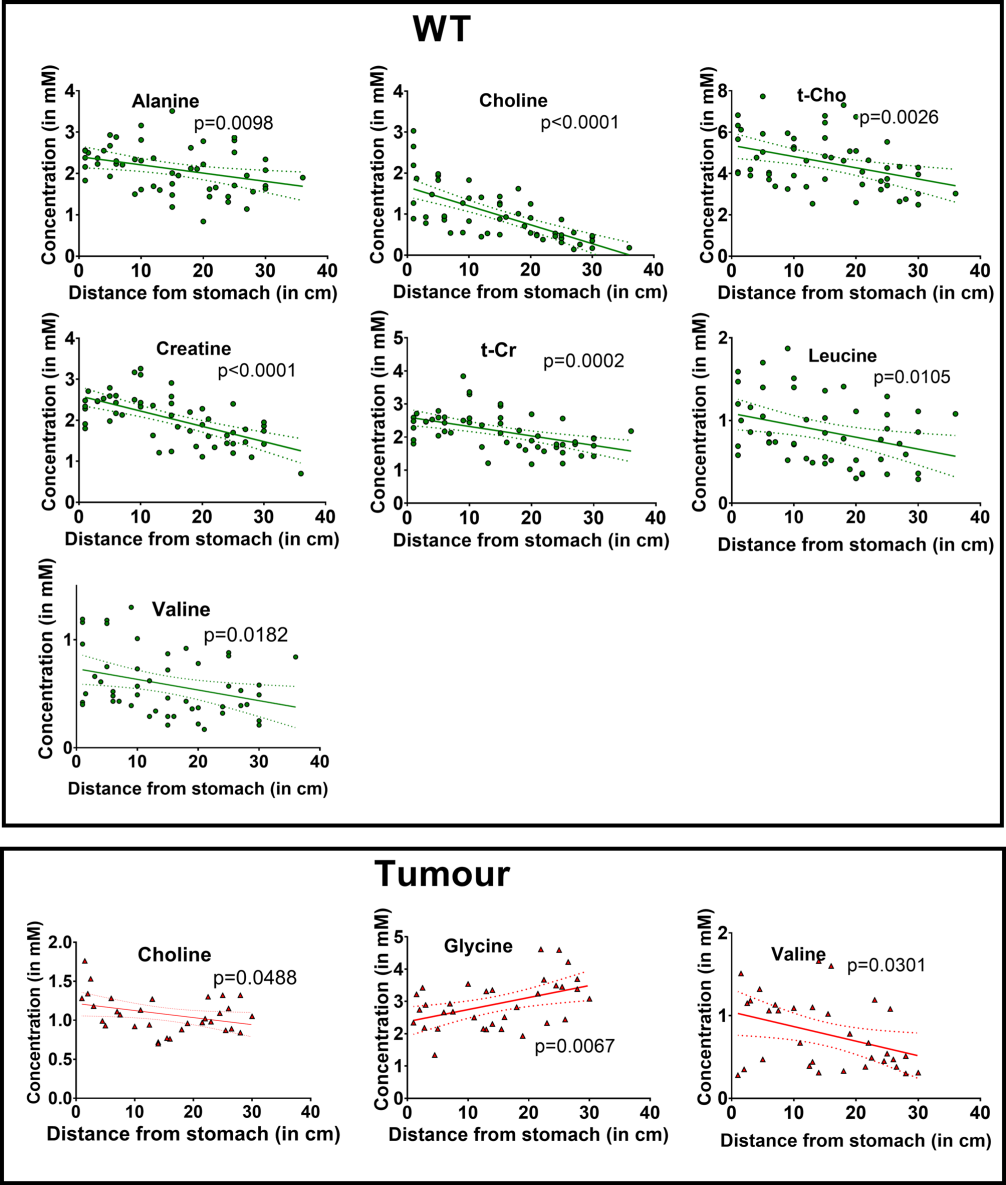
Metabolites that showed a significant concentration gradient in WT (green circles) and tumour (red triangles) tissue samples along the length of the mouse small intestine (see Table 1). The p values denote whether the gradient slope is significantly different from zero (*WT* wild-type C57BL/6J, *NAdj* normal tissue adjacent to ApcMin/+ tumours, *t-Cho* choline + phosphocholine + glycerophosphocholine, *t-Cr* creatine + phosphocreatine).

**Figure 2:**
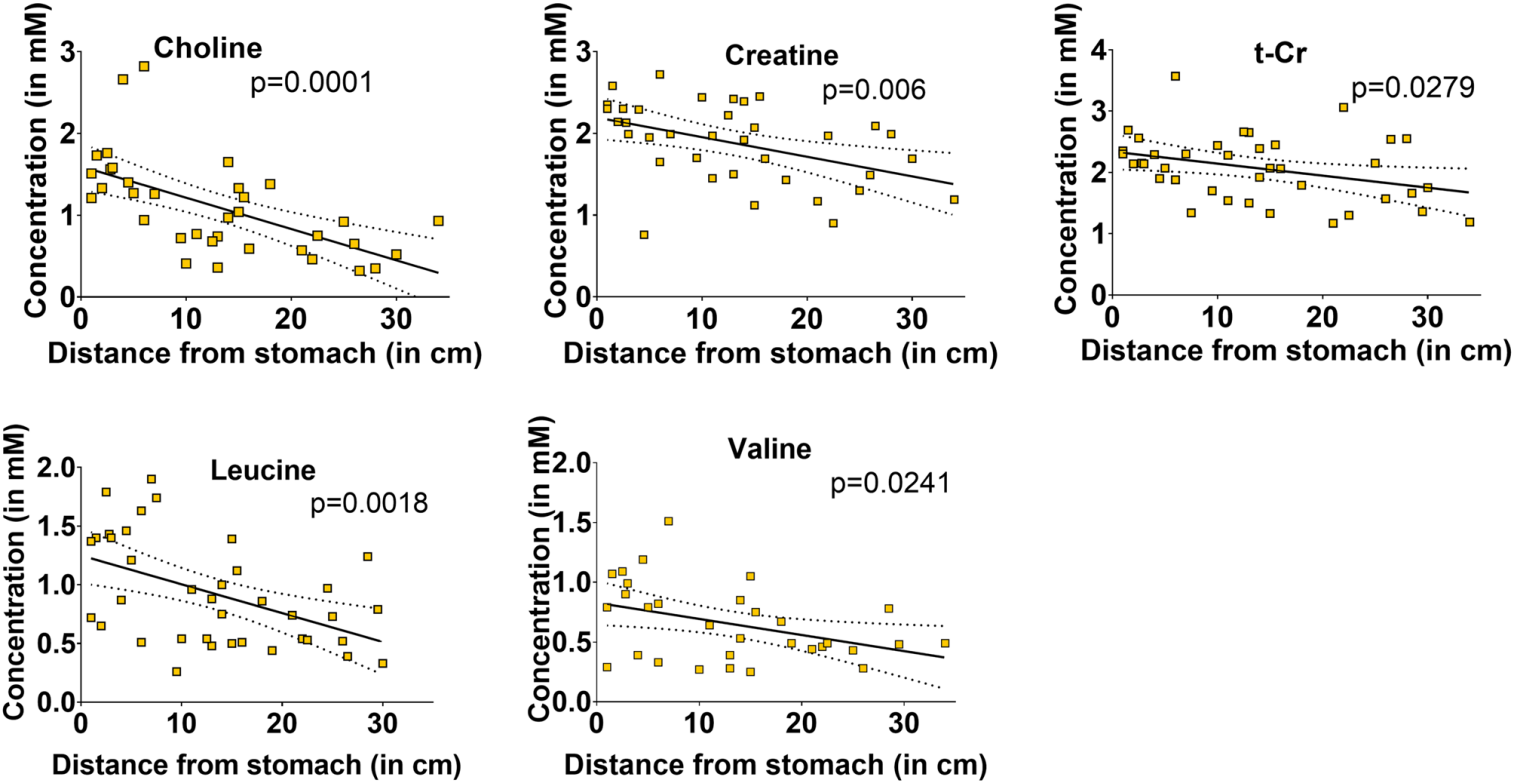
Metabolites that showed a significant concentration gradient in normal tissue adjacent to tumours along the length of the *Apc*^*Min/*+^ mouse small intestine (see Table 1). The p values denote whether the gradient slope is significantly different from zero (*t-Cho* choline + phosphocholine + glycerophosphocholine, *t-Cr* creatine + phosphocreatine).

The metabolite concentrations from wild-type C57BL/6J mice (WT), *Apc*^*Min/+*^ adenomatous tumours (Tumour) and from normal tissue adjacent to *Apc*^*Min/+*^ tumours (NAdj), plotted as a function of the distance of the sample from the pylorus, are shown in Figs. 1 and 2. Unexpectedly, the concentrations of several metabolites showed linear gradients throughout the length of the small intestine; these gradients were evident both in the intestinal tissues and in the tumours.

In the small intestines of WT mice there were statistically significant linear gradients from pylorus (high) to colon (low) in the concentrations of alanine, choline, t-Cho (choline + phosphocholine + glycerophosphocholine), creatine, t-Cr (creatine + phosphocreatine), leucine and valine (Table 1; Fig. 1). Five other metabolites, phosphocholine, glycerophosphocholine, glutamate, glycine and taurine, showed no significant gradients in the WT small intestine (Table 1).

**Table 1:** Metabolite concentration gradient slopes as a function of distance from the pylorus in wild-type mice (WT), normal tissue adjacent to *Apc*^*Min/*+^ tumours (NAdj) and Tumours.

Metabolite	WT		NAdj		Tumour	
	Slope	Is slope significantly non-zero? p values	Slope	Is slope significantly non-zero? p values	Slope	Is slope significantly non-zero? p values
Alanine	− 0.02	**0.0098**	NA	NA	NA	NA
Choline	− 0.046	** < 0.0001**	− 0.0383	**0.0001**	− 0.0091	**0.0488**
Phosphocholine	− 0.0074	0.4522	0.0127	0.235	− 0.0027	0.7327
Glycero− phosphocholine	0.0039	0.666	0.0285	0.0567	0.0079	0.7069
t-Cho	− 0.0543	**0.0026**	− 0.0064	0.7525	− 0.0083	0.6783
Creatine	− 0.0373	** < 0.0001**	− 0.024	**0.006**	0.0044	0.6887
t-Cr	− 0.0286	**0.0002**	− 0.0196	**0.0279**	0.0036	0.7023
Glutamate	0.0217	0.0574	0.0007	0.9493	0.0281	0.063
Glycine	− 0.002	0.7953	− 0.0176	0.2053	0.0372	**0.0067**
Taurine	0.0152	0.5038	− 0.0177	0.4637	− 0.0472	0.091
Leucine	− 0.0144	**0.0105**	− 0.0245	**0.0018**	− 0.0207	0.1024
Valine	− 0.0099	**0.0182**	− 0.0135	**0.0241**	− 0.0176	**0.0301**

Statistically significant gradients (p < 0.05) are shown in bold

*t-Cho* choline + phosphocholine + glycerophosphocholine, *t-Cr,* creatine + phosphocreatine, *NA* not applicable*.*

Tumour tissues from *Apc*^*Min/*+^ mice showed choline, glycine and valine concentration gradients, depending on the position of the tumour in the small intestine. The choline and valine gradients were negative, with the highest concentrations closest to the pylorus, like those in the WT tissue samples. Uniquely among the metabolites studied, however, glycine in Tumour samples showed a gradient with a *positive* slope along the small intestine from pylorus (low) to colon (high) (Fig. 1; Table 1); all other metabolites in Tumour and WT samples, showed negative slopes.

We also studied tissue samples from normal tissues adjacent to the tumours in *Apc*^*Min/*+^ mice (Fig. 2; Table 1). These NAdj samples showed all the metabolite gradients we had found in WT samples (Fig. 1) except for t-Cho, which did not have a significant gradient. Only choline and valine displayed concentration gradients in all three tissues—WT, NAdj and Tumour (Table 1).

We then used a linear regression model to determine whether the slopes of the gradients were significantly different when we compared the WT vs NAdj and the Tumour vs NAdj graphs (Figs. 1, 2; Supplementary Table S1). None of the slopes of the metabolite gradients were significantly different in WT compared to NAdj tissues (Figs. 1, 2; Supplementary Table S1), but the gradients of choline, creatine and glycine were significantly different between Tumour and NAdj samples (Figs. 1, 2; Supplementary Table S1 statistically significant gradients shown in bold red).

**Figure 3:**
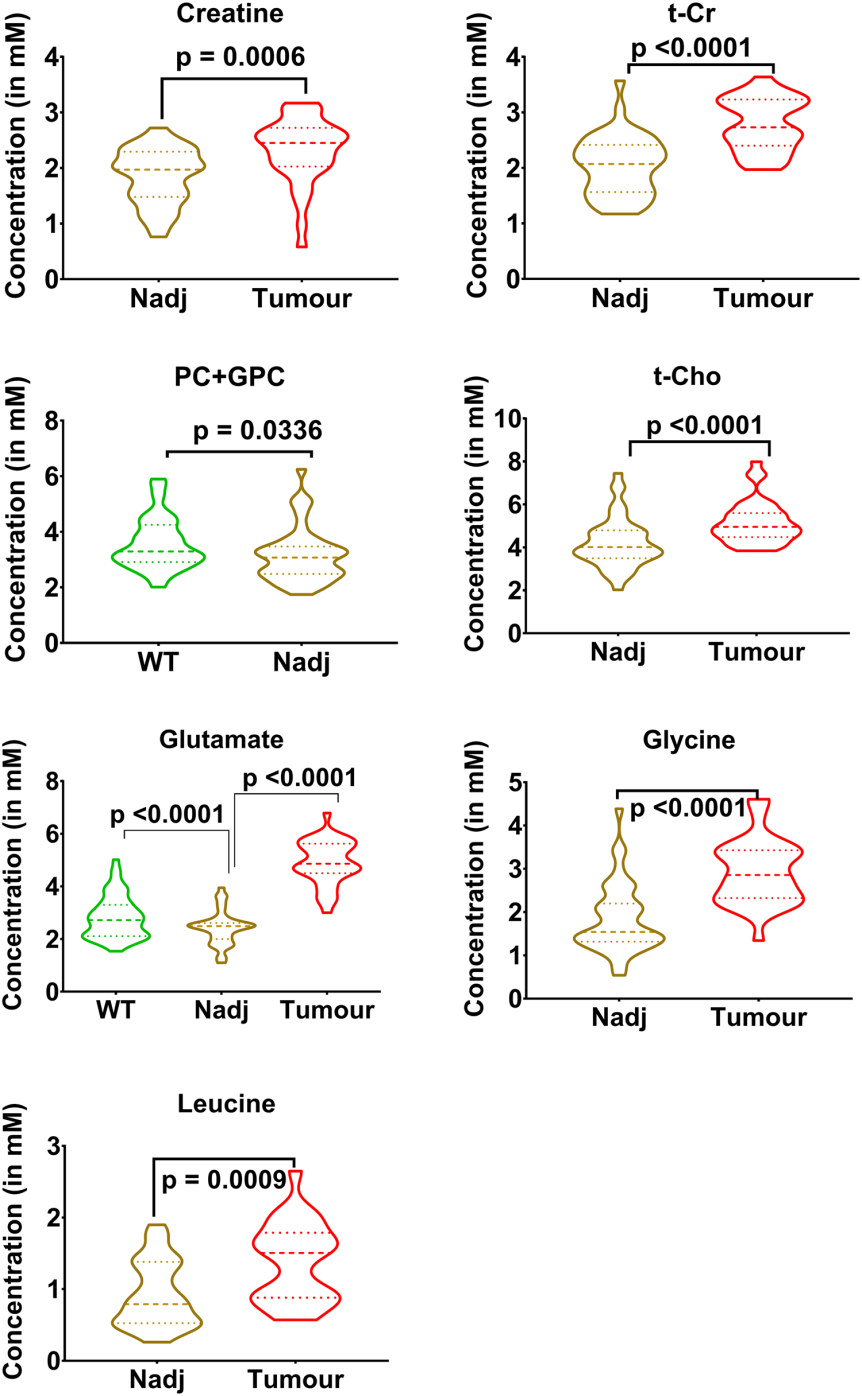
Metabolites that showed an overall significant difference in concentration between WT tissue (n = 56), normal tissue adjacent to tumours (NAdj, n = 43) and Tumour (n = 34) tissue samples (*WT* wild-type C57BL/6J, *NAdj* normal tissue adjacent to ApcMin/+ tumours, *t-Cho* choline + phosphocholine + glycerophosphocholine, *t-Cr* creatine + phosphocreatine. PC + GPC,phosphocholine + glycerophosphocholine).

In order to get an overview of the metabolomics of these samples, we also averaged the metabolite concentrations along the whole of the small intestine and then compared the mean metabolite values between the WT and NAdj samples and also between the Tumour and NAdj samples (Supplementary Table S3; Fig. 3). In general, the ratios of Tumour metabolite concentration to NAdj concentration were very similar for 5 of the metabolites, with a mean of 1.37 + 0.16 (t-Cho 1.17, creatine 1.24; t-Cr 1.36; glycine 1.58 and leucine 1.50). Only glutamate (2.03) had a markedly higher Tumour to NAdj ratio (Supplementary Table S3; Fig. 3). We also compared the mean metabolite concentrations from the WT and NAdj tissue samples and found that only glutamate was significantly different (Fig. 3). Another approach to quantifying metabolite concentrations is to use the intercept values from the linear regression fits, which indicate the mean metabolite contents at the pylorus. Even when there was no significant gradient, it was still sometimes possible to demonstrate a statistically significant intercept. For several metabolites (glycerophosphocholine, t-Cho, t-Cr, glutamate and leucine) the intercept at the pylorus was significantly higher in Tumour samples than NAdj samples (Supplementary Table S2; Supplementary Fig S3). Whenever the slope or the intercept of the linear fit of a metabolite was significantly different between Tumour and NAdj samples (Supplementary Table S2; Supplementary Fig. S3), the metabolite levels were consistently higher in the Tumour compared to NAdj samples (Supplementary Table S3; Fig. 3).

**Figure 4:**
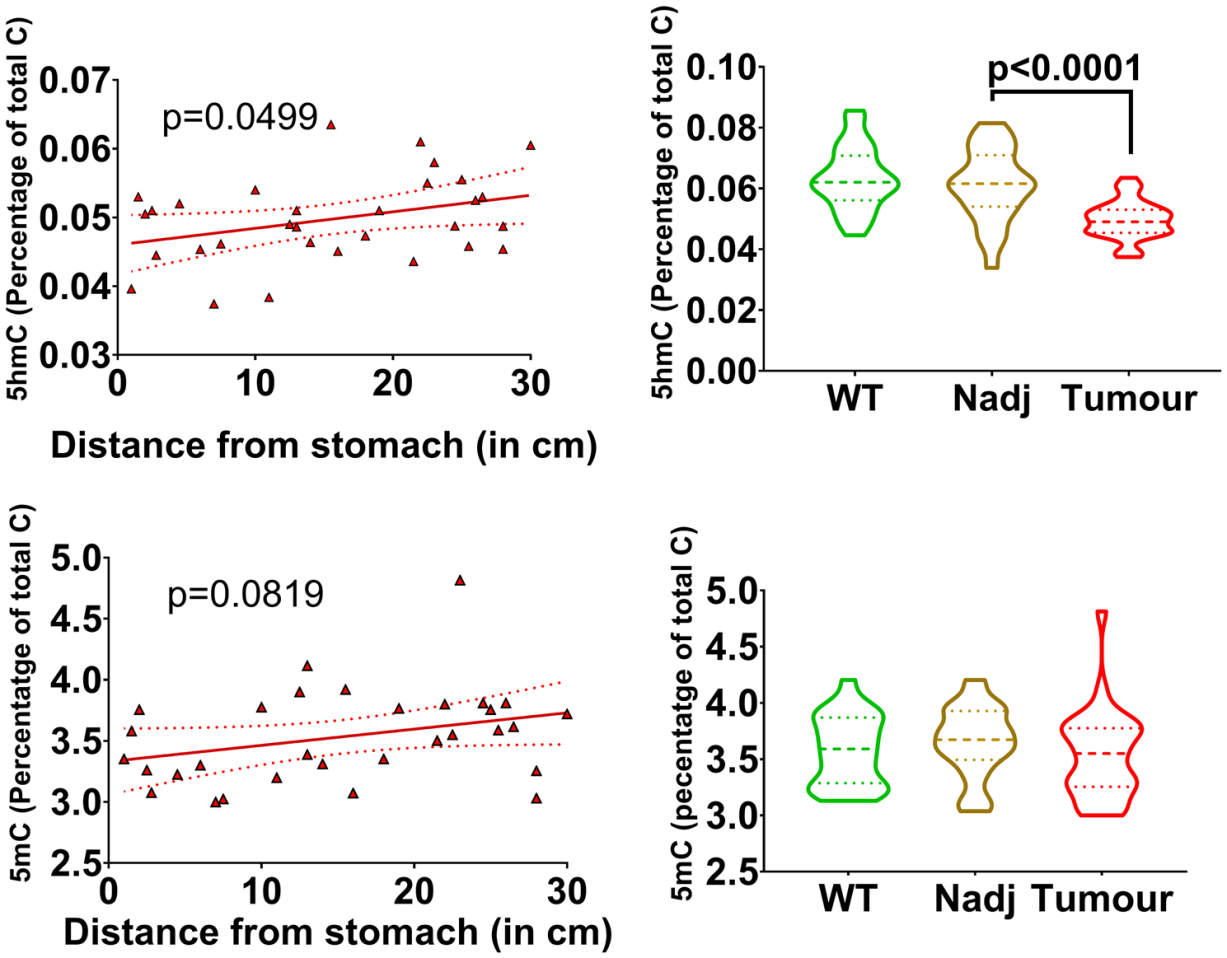
DNA cytosine hydroxymethylation (5hmC) and methylation (5mC) gradient slopes in tumours along the length of the small intestine. The p values denote whether the gradient slope was significantly different from zero (left column). Overall DNA cytosine modification levels by tissue (right column). DNA modification levels obtained in WT (n = 26), NAdj (n = 27) and Tumour (n = 31) tissues.

The 5-hydroxymethylcytosine (5hmC) and 5-methylcytosine (5mC) DNA modification levels, obtained by LC/MS, were also tested for topographical gradients. In the Tumour samples there were positive gradients from the pylorus for both 5mC and 5hmC (Fig. 4), but only the 5hmC slope was significant at the P < 0.05 level (Supplementary Table S4). The slopes of the 5hmC and 5mC gradients were not significantly different when comparing WT to NAdj, or NAdj to Tumour (data shown in Supplementary Tables S4, S5). The mean 5hmC and 5mC data were also tested for differences between the tissue types (Supplementary Table S4; Fig. 4). The overall 5hmC and 5mC levels were generally lower in Tumour compared to NAdj samples (Fig. 4), with a statistically significant difference for 5hmC.

## Discussion

The genetic and phenotypical similarities between *Apc*^*Min/*+^ mouse tumours and human intestinal tumours make this mouse a good model for studying human bowel cancer^12,13^. Two earlier metabolomic studies of *Apc*^*Min/*+^ mouse tumours focused on phenotype determination^7^ and the effects of a lipid-rich diet on tumour metabolism^14^. However, no systematic study has reported metabolite gradients along the small intestine of any mouse model.

Our own study was originally intended to test the hypothesis that metabolite concentrations along the length of the mouse small intestine are correlated with global DNA cytosine modifications, and that hypothesis turned out to be correct. However, we were also surprised to find that there were continuous, linear gradients of metabolite concentrations along the normal, WT mouse small intestine, which were to some extent reproduced in the intestinal tumours found at various positions in the small intestines of *Apc*^*Min/*+^ mice. The metabolite concentration gradients in the WT intestines were all negative, from a maximum at the pylorus to a minimum at the junction with the large bowel. Negative metabolite gradients were also observed in the tumours and the normal tissue adjacent to tumours, with the exception of glycine, which showed a positive gradient in the tumours themselves.

Another unexpected feature of these metabolite gradients was that they were always linear throughout the small intestine, rather than being different in the duodenum, jejunum and ileum, as had been found in human colon by Wang et al.^1^. There is also a report of varied epithelial gene and protein expression patterns along the human and mouse small intestines, with bicarbonate transporters prominent in the duodenum, where acidic stomach contents are neutralised, digestive enzymes and nutrient transporters highly expressed in the jejunum, and bile acid transporters present in the ileum^15^. However, Wright and Alison^11^ state that rodent small intestine does not show a distinct duodenum, jejunum and ileum, which could explain our observations of linear metabolic gradients rather than three discontinuous concentrations. Indeed, it has been known for many years that there are continuous anatomical gradients along the mouse small intestine^16^. The number of intestinal villi shrinks from more than 7,000 cells per villus at the pylorus to around 2,000 cells per villus at the distal end, and the number of cells per intestinal crypt falls from over 500 cells per crypt near the pylorus to 360 cells per crypt at the distal end. The crypt:villus ratio also falls from around 14 crypts per villus in the very proximal bowel to 6 crypts per villus at the distal end^16^. It seems possible, therefore, that these anatomical gradients are associated with some of the metabolic gradients that we observed in the WT small intestine, and perhaps also with those in Tumour and NAdj samples. However, some metabolites (phosphocholine, glycerophosphocholine, glutamate, glycine and taurine) showed no significant gradients in WT samples, and glycine in Tumour samples showed a positive gradient (Table 1), which would be difficult to explain on the basis of simple anatomical gradients. Another possibility is that the metabolic gradients we observed might be connected with the gradients in Wnt signalling and stem cell number found in mouse and human intestines^17^.

In general, fewer Tumour metabolites had concentration gradients than those in the WT or NAdj samples. One possible explanation would be that although the tumours originally arose from normal intestinal cells, and may initially have displayed the metabolic gradients we found in the normal small intestine, the transformed cells were then subjected to metabolic stresses associated with the malignant transformation itself (e.g. disruption of tight junctions and dedifferentiation), and with subsequent tumour growth, which could have selected tumour cells with genetic differences that over-rode some of the original metabolic gradients.

We also found generally higher metabolite concentrations in tumours than in the NAdj samples. That might be due to closer packing of the tumour cells, with e.g. less fat or fibrous tissue in the tumours than in the normal/healthy intestinal tissues. However, the concentrations of several metabolites (choline, phosphocholine, taurine and valine) showed no significant differences between the Tumour and NAdj samples, and glutamate was twice as high in the Tumour than in the NAdj samples, whereas the other significantly different metabolite concentrations were all in the range 23–58% (Supplementary Table S3), all of which argues against a simple anatomical explanation. Glutamate concentrations were also significantly different between the NAdj samples and the normal tissue in the same position in the WT small intestine (Fig. 3; Supplementary Table S3). As none of the other NAdj metabolite concentrations were significantly different from the WT concentrations (Supplementary Table S3) it seems possible that the high concentration of glutamate in the tumours was diffusing into the adjacent tissue. An alternative interpretation would be a field cancerisation effect, a process in which the cells in an area of normal tissue are all affected by carcinogenic alterations. Backshall et al. reported metabolic alterations in non-tumour gastrointestinal tissue of *Apc*^*Min/*+^ mice with a very low tumour burden, which they attributed to field cancerisation^7^.

Tumour samples showed a significantly positive glycine gradient along the small intestine (Fig. 1), which was the only positive metabolite gradient in any of the three tissues we examined. Increased tumour glycine concentrations have previously been found in human colon cancer^5^. Glycine can be formed from the glycolytic intermediate 3-phosphoglycerate, so it has been suggested that increased glycine in tumour tissues could result from a Warburg-like glycolytic phenotype in which cancer cells rely on anaerobic glycolysis even in the presence of adequate oxygen^5,18^. Glycine is also involved in the folate cycle, which supplies one-carbon units for de novo purine synthesis, so increased glycine could enhance nucleotide synthesis^19^.

Tumour glutamate was significantly higher than in NAdj samples, and higher in NAdj than in WT samples (Supplementary Table S3; Fig. 3). Glutamate, formed by glutaminolysis from glutamine, provides nitrogen for synthesis of nucleotides and proteins, which tumours need in large amounts. Increased glutaminolysis is therefore considered to be an emerging metabolic hallmark of cancer metabolism^20^. Glutamate undergoes transamination with pyruvate to form alanine, along with α-ketoglutarate which can enter the tricarboxylic acid cycle (TCA). Glycine, too, can enter the TCA via metabolism to pyruvate, as can leucine via metabolism to acetyl-CoA; all these metabolites can thus be energy sources or can form metabolites for synthesis of new cancer cells. Choline metabolites are involved in phospholipid metabolism and membrane turnover, and are often elevated in tumours, while phosphocreatine and creatine form a metabolic energy transfer system^21^. Thus the increased mean concentration levels of t-Cho, glycine, glutamate, leucine and t-Cr in *Apc*^*Min/*+^ mouse tumours compared to NAdj samples are consistent with metabolic reprogramming of phospholipid, amino acid and energy metabolism in the tumour tissue.

Cancer cell metabolism is also associated with epigenetic changes^11^. The Warburg effect can affect histone acetylation and cell proliferation through mechanisms mediated by butyrate^22^ and hydroxybutyrate^23^, whereas other metabolites including *S*-adenosyl methionine and 2-hydroxyglutarate are required for DNA methyltransferase (Dnmt) and hydroxylase activity^24^. However, we found no 5mC or 5hmC gradients in WT or NAdj tissue, suggesting that the metabolites measured in the small intestine may not limit Dnmt or TET activity. The absence of a gradient of 5hmC along the length of the healthy small intestine contrasts with that seen along the crypt-villus axis where the low levels of 5hmC in the proliferating progenitors at the crypt base are increased in the differentiated epithelium of the villus^8,25–27^. This indicates that the ratio of progenitor to differentiated cells remains constant along the length of the mouse small intestine.

The reduced 5hmC levels we found in the *Apc*^*Min/*+^ tumours, which are a hallmark of cancers^10,28–36^, are consistent with our previous findings in *Apc*^*Min/*+^ adenomas^8^ and colorectal cancer^37^ and more generally with 5hmC differences seen in any fast versus slow-proliferating healthy cells and tissues^10^. The shallow positive 5hmC gradient in Tumours seems to indicate relatively increased 5hmC levels in distal tumours, albeit levels remain lower than those in the adjacent normal tissue. The small 5hmC increase may be due to the serine, one-carbon cycle, glycine synthesis (SOG) pathway; this concomitantly produces a-ketoglutarate and glycine, which would fit with the positive glycine gradient in these tumours. However, the changes in glycine levels were not significantly correlated (R^2^ = 0.05) with 5hmC levels. We also tested for negative correlations between the 5hmC gradient and the negative gradients of choline and valine, but neither was statistically significant.

We found no evidence of reduced 5mC levels in Tumours relative to WT or NAdj samples. Recent genome-wide analyses have indeed shown equal gains and losses of methylation at regions with differential methylation between *Apc*^*Min/*+^ tumours and normal tissue^38^. In addition, we have previously shown that 5mC levels are progressively reduced from normal tissue to adenoma to adenocarcinoma^37^, suggesting that marked genome-wide changes observed with malignant progression have not taken place in the more benign *Apc*^*Min/*+^ intraepithelial adenomas.

## Conclusions

Normal WT mouse small intestine tissues showed continuous linear negative gradients of the metabolites alanine, choline, t-Cho, creatine, leucine and valine, with the highest concentrations in the intestinal tissues adjacent to the pylorus. Tumour tissue samples from *Apc*^*Min/*+^ mice showed similar negative gradients of choline and valine, but the glycine gradient was positive. The NAdj tissues showed negative gradients in the concentrations of choline, creatine, t-Cr, leucine and valine. There were also increased absolute levels of t-Cho, glycine, glutamate, leucine and t-Cr in the tumours when compared to NAdj tissue samples. These metabolic changes are consistent with increased glycolysis and modified phospholipid, amino acid and energy metabolism in the tumours. Further work will be necessary to investigate the metabolic origins of these gradients, which could, for instance, be connected with regional variations in active or passive uptake and secretion systems of the small intestine.

To our knowledge, this is the first report of measurements of global levels of DNA cytosine modification in the *Apc*^Min/+^ mouse small intestine. The levels of these epigenetic modifications were similar throughout the small intestine, and 5hmC was reduced in the proliferating cells and in tumours. Tumours, however, showed positive 5mC and 5hmC gradients from the pylorus, but only the 5hmC slope was statistically significant. These epigenetic gradients are likely due to metabolite/nutrient gradient(s) impacting cell proliferation rates. Our previous studies in cultured cells and mice^10^ have shown that 5hmC levels are highly dependent on the rate of cell proliferation (i.e., the average age of the DNA in the cell), as there is a significant time delay between DNA methylation during cell replication and 5mC oxidation. Further away from the pylorus there could be lower concentration of available nutrients, leading to fewer proliferating tumour cells and therefore higher average levels of 5hmC. It is also possible that gradients of metabolites directly affecting TET(s) enzymatic activity underly these epigenetic gradients. The TET(s)-mediated oxidation of 5mC to 5hmC can be modulated by the concentrations of 2-oxoglutarate, their substrate, or of the competitive inhibitors hydroxyglutarate, fumarate or succinate^39,40^. The levels of these TET-associated metabolites were below the detection threshold of our NMR method in the tissues we have analysed, therefore future lines of investigation would require the use of a more sensitive method such as mass spectrometry.

It would also be interesting to repeat earlier studies on rodents or humans that found discrete differences in mean metabolite concentrations between the duodenum, jejunum and ileum, in order to see whether these mean concentrations had actually been obtained from three regions of continuous gradients in the metabolite concentrations.

## Methods

### Animals and sample collection

*Apc*^*Min/*+^ mice were bred and maintained by backcrossing male *Apc*^*Min/*+^ mice with females from a colony of C57BL/6J mice. Tissue samples were taken from normal tissue of wild-type C57BL/6J mice (WT), *Apc*^*Min/*+^ adenomatous tumours (Tumour) and from normal tissue adjacent to *Apc*^*Min/*+^ tumours (NAdj). Tissues were dissected under a variable-magnification stereo dissecting microscope, at various distances from the pylorus (i.e. the junction with the stomach). WT samples were taken at approximately 2.8 cm (range 2–4 cm) intervals. In the *Apc*^*Min/*+^ mice, both tumour and Nadj sampling was done according to the tumour location, which resulted in an average sampling distance of 2.6 cm (range 1–6 cm). Sample weights ranged from 2 to 12 mg, with the smaller samples coming from small adenomas. Samples from the small intestine were a mixture of epithelial and lamina propria cell types. The study approach for sample dissection and analysis is shown in Supplementary Fig. S1. Anatomical sites within the mouse small intestine were measured as the distance from the pylorus, as advised by Wright and Alison^39^.

### NMR spectroscopy

HR-MAS ^1^H NMR of WT, NAdj and Tumour samples was performed on a Bruker 600 MHz instrument, with a 4 mm HRMAS probe, operated at a spin rate of 3000 Hz and a sample temperature of 4 °C. LCModel software with a modified basis set was used on water-suppressed spectra to estimate the metabolite concentrations^40^. Since these were not brain tumours, *N*-acetyl aspartate and *N*-acetyl aspartyl glutamate were omitted from the analysis. A phosphocreatine signal was simulated in the basis set, since phosphocreatine was present in the samples. Absolute metabolite concentrations were quantified relative to the water signal observed in each individual experiment^40^. This methodology was validated with phantoms containing known concentrations of metabolites. NMR data were then obtained from WT (35 samples from 3 animals) and *Apc*^*Min/*+^ (43 NAdj and 34 Tumour samples, from 3 animals) tissue samples from mouse small intestines, taken at numerous different distances from pylorus to colon. This procedure took 5–10 min per intestine, so we performed a separate experiment in which tissues (n = 21 samples from 3 animals) were sampled in the opposite direction, from colon to stomach in order to assess possible sampling errors due to tissues that were sampled last being subjected to a longer period of ischemia. There was evidence of elevated lactate concentrations in some of the samples that had undergone the longest period of ischaemia before freezing, so we have not reported any lactate results. None of the other metabolites measured showed any difference in concentration in the samples that had been sampled in the reverse direction (Supplementary Fig S2).

In many ex vivo MRS studies, the creatine and choline region signals are quantified as total-creatine and total-choline, due to poor resolution of the several metabolite signals in these clusters. We too found that the phosphocreatine signals at 3.03 ppm were not consistently observed or resolved from creatine in the HRMAS ^1^H NMR spectra of the tissue samples. Even in samples where phosphocreatine signals were credibly resolved (3 Nadj samples), we have therefore added the concentrations of creatine and phosphocreatine together and referred to the sum as total creatines (t-Cr). Similarly, the choline, phosphocholine and glycerophosphocholine peaks in the 3.20–3.23 ppm spectral region could not be resolved in 3 WT, 5 NAdj and 3 Tumour samples, whereas in other spectra they were very well resolved. For all samples therefore, we have summed the concentrations of choline, phosphocholine and glycerophosphocholine as total choline (t-Cho). However, when the individual peaks of the cholines and creatines could be resolved we have also quantified them separately.

### Mass spectrometry

Liquid chromatography/mass spectrometry (LC/MS) was performed on DNA extracted from tissues recovered after NMR analysis (WT n = 27, NAdj n = 30 and Tumour n = 30) to measure global levels of 5-methylcytosine (5mC) and 5-hydroxymethylcytosine (5hmC) using methods previously described^10,37^.

### Statistical analysis

All statistical analyses were performed with GraphPad Prism software. Linear regression models were fitted for each individual metabolite or DNA modification, to investigate whether metabolite concentrations and DNA modifications were linearly dependent on distance from the pylorus, and whether this varied between WT, NAdj and Tumour samples. The comparisons of interest were NAdj versus WT and NAdj versus Tumour.

Linear regression was performed for each metabolite or DNA modification to test for varying intercepts and slopes. Where the intercept p value has not been entered (NA), it indicates that the model allows for varying slopes only, and that there were no significant differences in the intercept. Otherwise, the models allow for varying intercepts and slopes.

### Ethics approval

All experiments were performed under UK Home Office License PPL 80/2427 and all procedures were approved by the CRUK Cambridge Institute Animal Welfare and Ethical Review Body.

## Supplementary information

Supplementary information

## Data Availability

The datasets generated during and/or analysed during the current study are available from the corresponding author on reasonable request.
