# Supplementary material for: ApcMin/+ tumours and normal mouse small intestines show linear metabolite concentration and DNA cytosine hydroxymethylation gradients from pylorus to colon
Source: Sci Rep. 2020 Aug 12;10:13616. doi: 10.1038/s41598-020-70579-w (PMC7423954; doi:10.1038/s41598-020-70579-w)

# *Apc*$^{Min/+}$ tumours and normal mouse small intestines show linear metabolite concentration and DNA cytosine hydroxymethylation gradients from pylorus to colon

**Basetti Madhu, Santiago Uribe-Lewis, Martin Bachman, Adele Murrell and John R Griffiths**

## Supplementary Material

**Table S1. Statistical significance of slopes of metabolite gradients between tissues.** WT = wild-type C57BL/6J, NAdj = normal tissue adjacent to *Apc*$^{Min/+}$ tumours. t-Cho, choline + phosphocholine + glycerophosphocholine; t-Cr, creatine + phosphocreatine.

| Metabolite | WT vs NAdj slopes<br>p-value | Tumour vs NAdj slopes<br>p-value |
|---|---|---|
| **Choline** | 0.4828 | **0.0053** |
| **Phosphocholine** | 0.1779 | 0.2538 |
| **Glycero-phosphocholine** | 0.1329 | 0.4096 |
| **t-Cho** | 0.0757 | 0.9489 |
| **Creatine** | 0.2066 | **0.0382** |
| **t-Cr** | 0.4194 | 0.0723 |
| **Glutamate** | 0.1963 | 0.1305 |
| **Glycine** | 0.2855 | **0.0052** |
| **Taurine** | 0.3301 | 0.4175 |
| **Leucine** | 0.2604 | 0.7754 |
| **Valine** | 0.6008 | 0.6676 |

**Table S2. Statistical significance of Y-intercepts of metabolite gradients between tissues.** WT = wild-type C57BL/6J, NAdj = normal tissue adjacent to *Apc*$^{Min/+}$ tumours. t-Cho, choline + phosphocholine + glycerophosphocholine; t-Cr, creatine + phosphocreatine.

| Metabolite | Y-intercept | | | | |
|---|---|---|---|---|---|
| | WT | NAdj | WT vs Nadj p-value | Tumour | Tumour vs Nadj p-value |
| Choline | 1.66 | 1.60 | 0.6724 | 1.22 | N/A |
| Phosphocholine | 1.71 | 1.20 | 0.0956 | 1.57 | 0.22 |
| Glycero-phosphocholine | 1.94 | 1.58 | 0.9147 | 2.50 | **0.0082** |
| t-Cho | 5.36 | 4.26 | 0.1110 | 5.27 | **0.0004** |
| Creatine | 2.60 | 2.19 | **0.0262** | 2.28 | N/A |
| t-Cr | 2.61 | 2.34 | 0.1903 | 2.71 | **<0.0001** |
| Glutamate | 2.49 | 2.41 | **0.0176** | 4.24 | **<0.0001** |
| Glycine | 1.76 | 2.12 | 0.3397 | 2.38 | N/A |
| Taurine | 6.34 | 7.39 | 0.0740 | 7.59 | 0.4976 |
| Leucine | 1.09 | 1.25 | 0.7884 | 1.65 | **0.0003** |
| Valine | 0.73 | 0.83 | 0.4637 | 1.04 | 0.076 |

**Table S3. Mean concentrations of metabolite in wild type C57BL/6J tissues (WT), normal tissue adjacent to *Apc*$^{Min/+}$ tumours (NAdj) and *Apc*$^{Min/+}$ Tumour tissue.** t-Cho, choline + phosphocholine + glycerophosphocholine; t-Cr, creatine + phosphocreatine.

| | WT V NAdj | | | NAdj vs Tumour | | |
|---|---|---|---|---|---|---|
| | WT | NAdj | | NAdj | Tumour | |
| **Metabolite** | **Mean ± 1 s.e.m.** | **Mean ± 1 s.e.m.** | **t-test** | **Mean ± 1 s.e.m.** | **Mean ± 1 s.e.m.** | **t-test** |
| **Choline** | 0.959± 0.086 | 1.085± 0.093 | 0.3339 | 1.085± 0.093 | 1.081± 0.050 | 0.9649 |
| **Phosphocholine** | 1.564± 0.091 | 1.377± 0.095 | 0.1711 | 1.377± 0.095 | 1.532± 0.069 | 0.2110 |
| **Glycerophosphocholine** | 1.965± 0.090 | 1.973± 0.133 | 0.9581 | 1.973± 0.133 | 2.674± 0.187 | 0.0025 |
| **t-Cho** | 4.519± 0.177 | 4.204± 0.178 | 0.2196 | 4.204± 0.178 | 5.158± 0.171 | 0.0003 |
| **Creatine** | 2.025± 0.078 | 1.849± 0.081 | 0.1321 | 1.849± 0.081 | 2.309± 0.101 | 0.0006 |
| **t-Cr** | 2.168± 0.075 | 2.044± 0.083 | 0.2787 | 2.044± 0.083 | 2.781± 0.081 | <0.0001 |
| **Glutamate** | 2.819± 0.108 | 2.423± 0.102 | 0.0120 | 2.423± 0.102 | 4.906± 0.144 | <0.0001 |
| **Glycine** | 1.731± 0.0705 | 1.846± 0.126 | 0.3999 | 1.846± 0.126 | 2.924± 0.129 | <0.0001 |
| **Taurine** | 6.542± 0.213 | 7.111± 0.216 | 0.0692 | 7.111± 0.216 | 6.808± 0.253 | 0.3630 |
| **Leucine** | 0.8692± 0.055 | 0.922± 0.075 | 0.5621 | 0.922± 0.075 | 1.38± 0.113 | 0.0009 |
| **Valine** | 0.5886± 0.041 | 0.650± 0.055 | 0.3637 | 0.650± 0.055 | 0.777± 0.074 | 0.1764 |

**Table S4. Statistical significance of DNA cytosine hydroxymethylation (5hmC) and methylation (5mC) slopes by tissue.** 5hmC = hydroxymethylation, 5mC = methylation, WT = wild-type C57BL/6J, NAdj = normal tissue adjacent to Tumour in *Apc*$^{Min/+}$.

| | 5hmC | | | 5mC | | |
|---|---|---|---|---|---|---|
| | *WT* | *NAdj* | *Tumour* | *WT* | *NAdj* | *Tumour* |
| **Slope** | -0.0001 | 0.0004 | 0.0002 | -0.0008 | -0.0014 | 0.0133 |
| **Is slope significantly non-zero? P-values** | 0.6017 | 0.0756 | **0.0499** | 0.8884 | 0.8224 | 0.0819 |

**Table S5. Statistical significance of DNA cytosine hydroxymethylation (5hmC) and methylation (5mC) slopes between tissues.** WT = wild-type C57BL/6J, NAdj = normal tissue adjacent to Tumour in *Apc*$^{Min/+}$.

| | *WT vs NAdj* | | | *NAdj vs Tumour* | | |
|---|---|---|---|---|---|---|
| | WT slope | NAdj slope | p-value | NAdj slope | Tumour slope | p-value |
| **5hmC** | -0.0001 | 0.0004 | 0.0869 | 0.0004 | 0.0002 | 0.4429 |
| **5mC** | -0.0008 | -0.0014 | 0.9485 | -0.0014 | 0.0133 | 0.1373 |

**Fig S1. Method for obtaining samples for measurements of metabolite concentrations and DNA modifications.** An example of a small intestine whole-mount with the gastro-duodenal junction at the top of the left hand mount and the caecum and colon in the right hand mount. The caecum and colon (far right) were not assessed. Tissues from WT and *Apc*$^{Min/+}$ animals were dissected at measured distances from the stomach, and were then used for metabolite and DNA modification analyses.

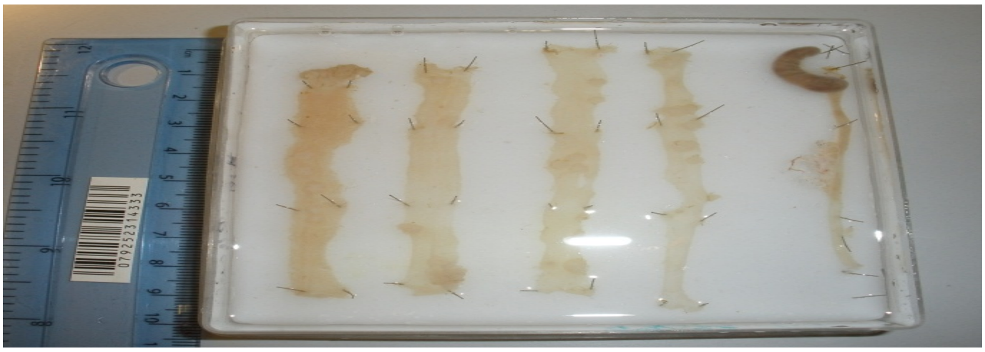

↓ Dissect normal tissues and tumours.

***HR-MAS $^{1}$H NMR***

↓ Recover tissues (where possible), extract DNA.

***LC-MS***

**Fig S2. Plot of metabolite concentrations in WT gut tissues as a measure of tissue dissection order.** Small intestine tissues were dissected proximal to distal from the pylorus (top to bottom) or distal to proximal (bottom to top) to assess whether metabolite concentrations would be dependent on time to snap freeze in liquid nitrogen. PC, phosphocholine; GPC, glycerophosphocholine.

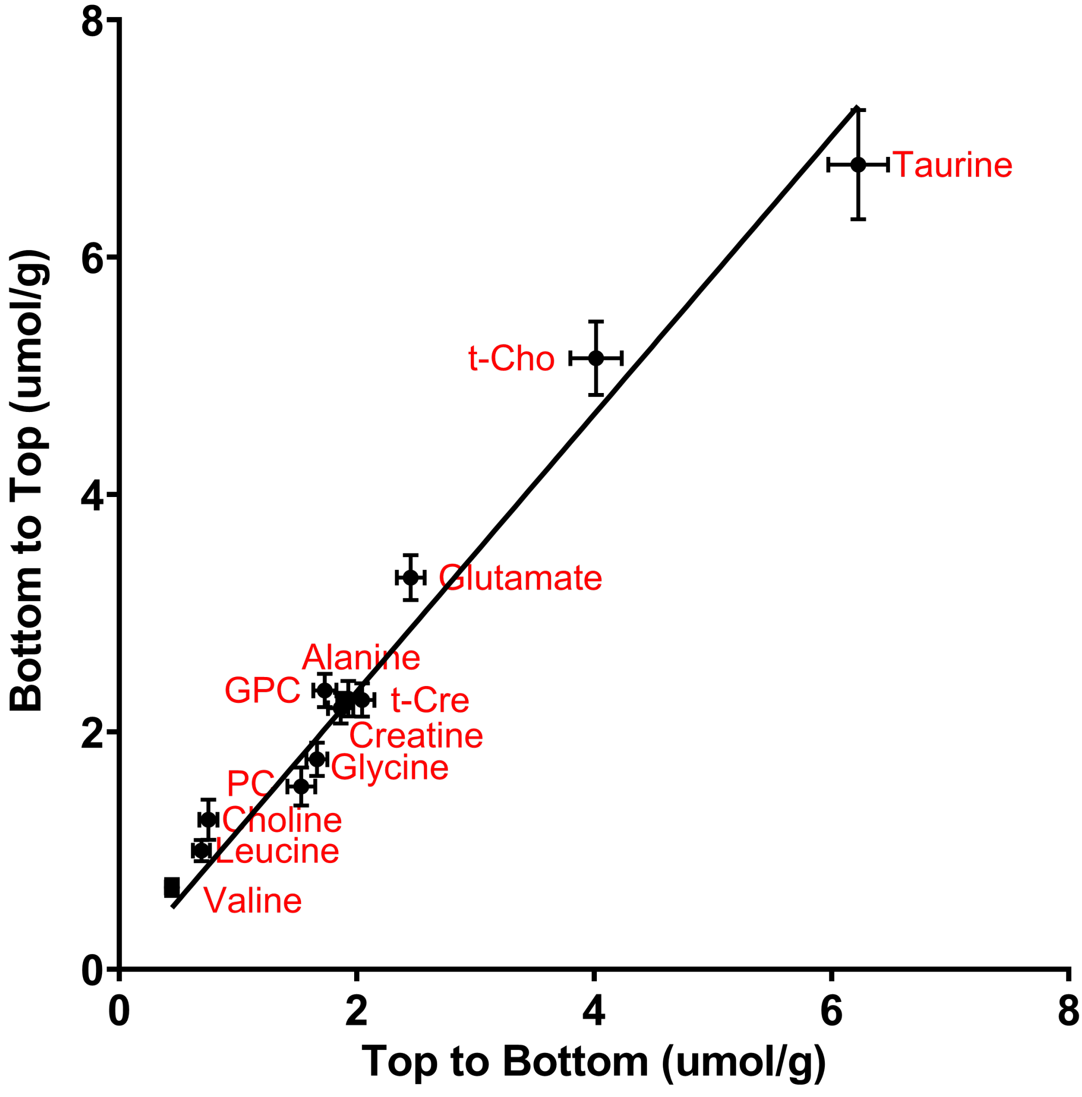

**Fig S3. Plot of metabolite concentrations in normal tissue adjacent to tumours (NAdj) and Tumour tissues along the small intestine as a function of distance from pylorus. t-Cho, choline + phosphocholine + glycerophosphocholine; t-Cr, creatine + phosphocreatine.**

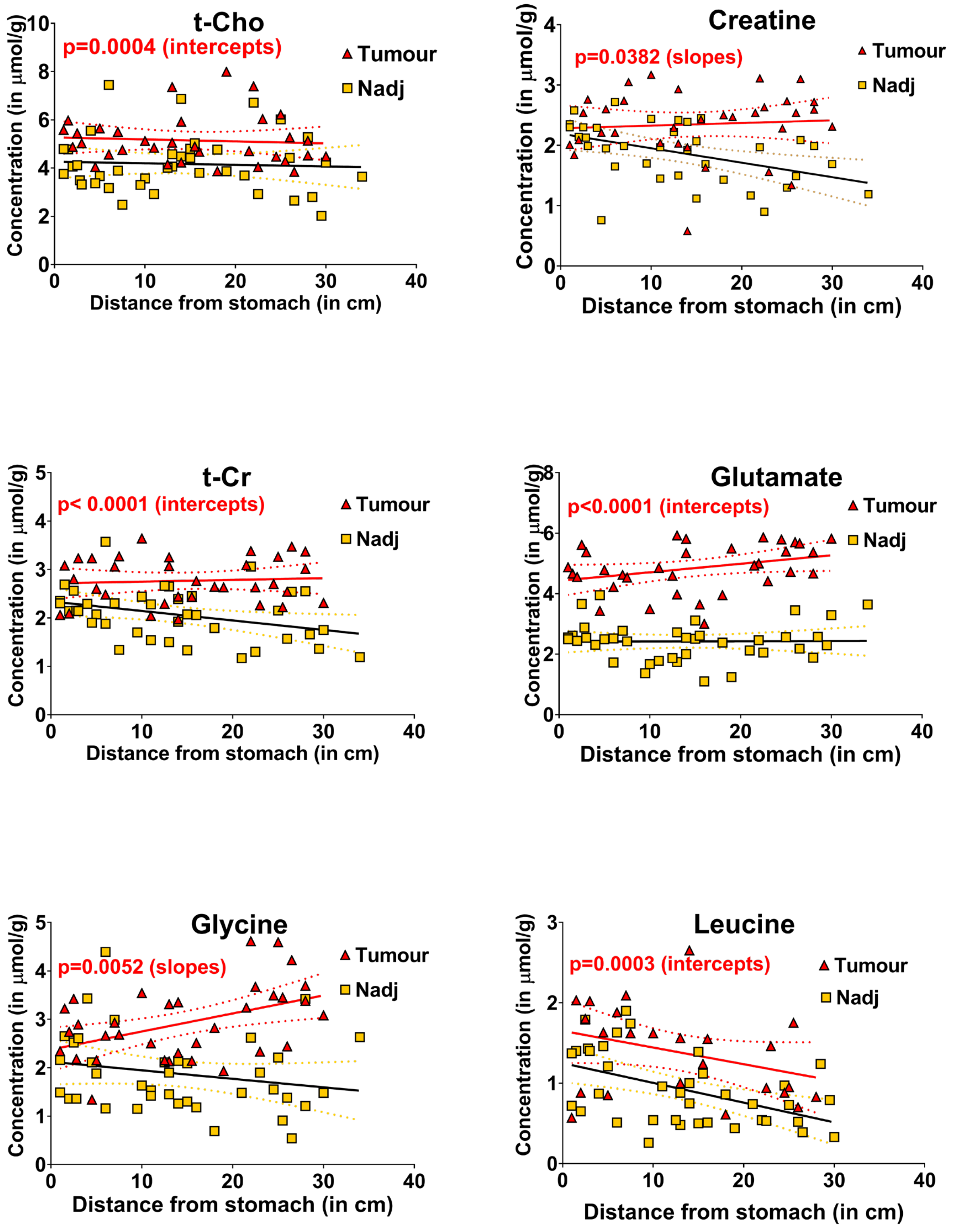

Supplement: Supplementary file 1 — Supplementary information [file 41598_2020_70579_MOESM1_ESM.pdf]
